# The influence of survivin shRNA on the cell cycle and the invasion of SW480 cells of colorectal carcinoma

**DOI:** 10.1186/1756-9966-27-20

**Published:** 2008-07-18

**Authors:** Liu Zhonghong, Lin Lianjie, Zheng Changqing, He Ying, Jin Yu, Lin Yan

**Affiliations:** 1 Department of the Digestive Internal Medicine, Shengjing Hospital of China Medical University, Shenyang, PR China

## Abstract

**Background:**

The objective was to understand the influence of Survivin plasmid with short hairpin RNA (shRNA) on the cell cycle, invasion, and the silencing effect of Survivin gene in the SW480 cell of colorectal carcinoma.

**Methods:**

A eukaryotic expression vector, PGCH1/Survivin shRNA, a segment sequence of Survivin as target, was created and transfected into colorectal carcinoma cell line SW480 by the non-lipid method. The influence on the Survivin protein was analyzed by Western blotting, while the cell cycle, cell apoptosis were analyzed by flow cytometry, and invasion of the cell was analyzed by Transwell's chamber method.

**Results:**

After the transfection of PGCH1/Survivin shRNA, the expression of Survivin protein in SW480 cells was dramatically decreased by 60.68%, in which the cells were stopped at G2/M phase, even though no apoptosis was detected. The number of transmembranous cells of the experimental group, negative control group, and blank control group were 14.46 ± 2.11, 25.12 ± 8.37, and 25.86 ± 7.45, respectively (P <*0.05*).

**Conclusion:**

Survivin shRNA could significantly reduce the expression of Survivin protein and invasion of SW480 cells. Changes in cell cycle were observed, but no apoptosis was induced.

## 1. Introduction

Colorectal cancer is one of the most commonly seen, malignant tumors in human, and the incidence rate is gradually increasing year by year. In United States, it was the second reason leading to death caused by malignant tumor, and the number of annual incidence had reached 135,000 people. Currently, combined therapy, which primarily focused on surgical removal, is employed for most cases of colorectal cancer. However, the cost for this combined treatment is relatively higher and more side effects existed. Also, the primary reasons for failed therapy are the localized reoccurrence and hepatic metastasis. Therefore, to cure patients in time, prevention and early detection of localized reoccurrence and hepatic metastasis, as well as screening of high-risk patients and prediction of reoccurrence and metastasis, have significant meaning in improving patient's life quality and survival rate. With the development of molecular biology and genetic engineering, the gene therapy is the research focus for prevention and treatment of tumor. Currently, gene therapies for tumor include gene replacement, antisense nucleic acid technique, cytokine gene therapy, and RNA interference technique mostly focused in recent years. RNA interference is the most effective gene silencing technique currently, while being simple, effective, and specific as its advantages. It primarily uses nuclease to cut double-stranded RNA (dsRNA) into 21 to 25 nt of small interfering RNA, also known as siRNA. The siRNA, based on the principle of base pairing, could specifically recognize and cleave the homogenous target mRNA molecule, to achieve the silencing effect. The short hairpin RNA (shRNA) could automatically be processed to become siRNA to silence gene, and it was proven to be more stable than siRNA. Survivin is a member of apoptotic inhibitory protein, and is selectively expressed in tumor tissues, especially with high expression in colorectal cancer, rather than in normal mature tissues. [1,2]. Survivin gene is an ideal target site for treating colorectal tumor [3]. In this study, a plasmid with expression of shRNA, which targeted specifically the Survivin gene, was created and used to transfect SW480 cells. The influence of Survivin shRNA on the cell cycle and the invasion ability of SW480 cells of colorectal carcinoma, as well as its efficacy in silencing Survivin gene, were analyzed in this paper.

## 2. Materials and methods

### Reagents and equipments

The pGCsilencer™H1/Neo/GFP/RNAi expression vector was purchased form Genechem Company. Restriction endonuclease Hind and BamH1 were from Qiagen Company. DMEM culture media were from GIBCO Company. Fetal bovine serum was purchased from Tianjing. Survivin polyclonal antibodies were purchased from Santa Cruz Company. FuGENE6, non-lipid transfection reagent, was product of Roche Company. Human colon carcinoma cell line SW480 was from Nanjing Kaiji Biology. Transwell's chamber (8 μm of pore diameter) was product of Millipore Company. Artificial basal membrane known as Matrigel was purchased from BD Company.

### Design of target sequence of shRNA

Survivin gene sequence came from GenBank (NM001168), with the size of 999 bp. Usage of GeneChem™siRNA, based on the design principle for siRNA, 19nt target sequence of siRNA was selected as template: GGCTGGCTTCATCCACTGC (86–104). By BLAST analysis, it was no homogy with other coding sequences in human. The 5'-end of the sequence corresponded with the cut-off point for BamHI enzyme, while the 3'-end, which had the T6 sequence, corresponded to the cutting site for Hind III enzyme. A ring sequence of 9 base pairs existed between the sense and antisense strands (TTCAAGAGA). The pGCsi-H1/Neo/GFP/NON RNAi vector was used as negative control, which did not interfere the studied intrinsic gene.

### The construction of pGCH1/Survivin shRNA expression plasmid

Two strands of oligonucleotides would undergo annealing, double enzyme-cut by restriction endonuclease Hind and BamH1, ligation, and transformation, as well as the PCR process, to identify positive clones. The constructed pGCH1/Survivin shRNA expression plasmid would be sequencing by Shanghai Invitrogen Biotechnology Company.

### Cell culture and transfection

#### Grouping

Blank control group, negative control group, and specific interference group were set up in this experiment.

#### Transfection

The human colon cancer cell line SW480 was recovered in water bath and was cultured in DMEM media with 10% fetal bovine serum, 100 U/ml penicillin, and 100 μg/ml streptomycin at 37°C incubator containing 5% CO_2_. One day before the transfection, cells in logarithmic growth phase were digested by 0.25% trypsin. Cells were seeded into the 6-well plate with 3 × 10^5 ^cells per hole. After the cells adhered to the bottom, they were transfected by following the instructions of the test kit.

### Measurement Survivin protein level in each group by Western blotting

After 48 hours of transfection, cells and supernatant of each group would be collected. Proteins were extracted after break-down of cells by SDS boiling method. Proteins were quantified by Bradford method. 40 μg of protein underwent SDS-PAGE and was transferred to PVDF membrane afterward. It was then sealed at room temperature for 2 hours. The primary antibodies, rabbit anti-human Survivin antibodies, were added at a ratio of 1:1000, and incubated overnight at 4°C. The membrane was washed with PBS. Then, the secondary antibodies, goat anti-rabbit IgG/HRP antibodies, were added at a ratio of 1:4000, and incubated at room temperature for 2 hours. The membrane was washed three times and reacted with chemiluminescent agent for 5 minutes. It was then ECL tabletting, exposed, and displayed.

### Measurment the cell cycle and apoptosis of SW480 cells by flow cytometry

After 48 hours of transfection, cells of each group were digested by Trypsin. Afterward, pre-cooled PBS in 4°C was added to prepare it into single-cell suspension and centrifuged for 10 minutes at 1000 rpm. The supernatant was discarded. 0.5 ml of pre-cooled 70% alcohol was then added and the solution was left in 4°C refrigerator overnight. The solution was then centrifuged again for 10 minutes at 1000 rpm and the supernatant was discarded. The remaining cells were then re-suspended by PBS and centrifuged. The supernatant was discarded. Afterward 500 μl of PBS was added to re-suspend it again. 500 μl PI staining solution was added. It was left on ice, and incubated away from light for 30 minutes. The cell cycle and apoptosis were then measured by flow cytometry.

### Analysis of cell invasion ability

On the side, facing the upper chamber, of the covering membrane of the Transwell's chamber, 30 μg Matrigel was applied. The lower chamber was added with 400 ul of conditioned medium from normal fibroblasts NIH3T3 cells(please note to avoid production of air bubbles between the culture media and the small chamber). The upper levels were respectively added with 100 ul of single-cell suspension (1 × 10^6^) of each group, which was already transfected for 48 hours.

#### Cell culturing

Placed in an incubator of 37°C and 5% CO_2_, cells were cultured for 48 hours.

#### Statistics of results

The small chamber was taken out and the non-migrated cells and Matrigel in the upper room of chamber were carefully removed by wool. It was then fixed at constant temperature for 30 minutes with 90% ethanol, and stained with 0.1% crystal violet solution for 20 minutes. It was placed under upright microscope for observation and photography. Randomly, 10 visual fields were selected and counted of penetrating cells. The average was calculated, in which the relative number of transmembrane cells represented the invasion ability of tumor cells. For each specimen, it was repeated twice, with the experiment also repeated for three times. Invasion inhibition rate = (1 – number of penetrating cells in experimental group/number of penetrating cells in control group) × 100%.

### Statistical analysis

The SPSS 13.0 software was used to establish database for statistical analysis. The number of transmembrane cells was represented in form of $\bar{\text{x}}$ ± *s*. The inter-group differences were using single-factor variance analysis, where *p *value less than 0.05 was considered as statistical significance.

## 3. Results

### The construction of pGCH1/Survivin shRNA expression plasmid

**Figure 1:**
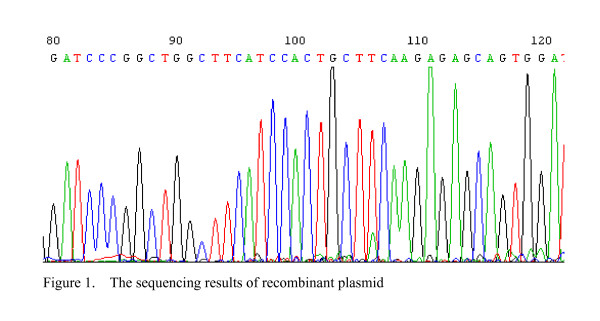
The sequencing results of recombinant plasmid.

**Figure 2:**
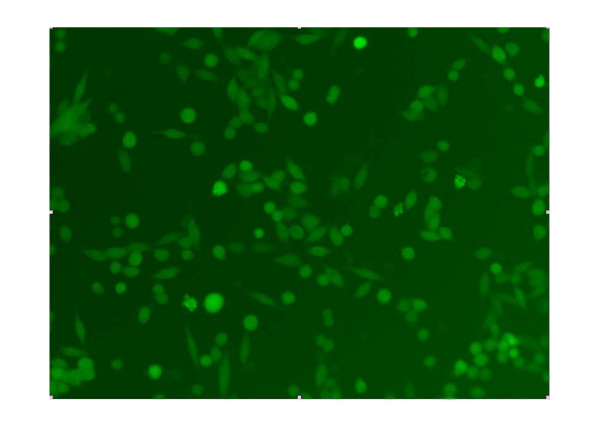
Observation of the expression of green luminescent protein in SW480 cells transfected with pGCH1/Survivin shRNA under fluorescent microscope.

The sequencing result showed the shRNA coding sequence in recombinant plasmid was exactly coincidence with our designed target Survivin's nucleic acid sequence. It suggested that the construction of recombinant plasmid pGCsiRNA-Survivin was a success (Figure 1). After the colorectal cancer cell line SW480 was transfected with the plasmid, cells showed green luminescence, suggesting the correct expression of pGCH1/Survivin shRNA (Figure 2).

### The influence of pGCH1/Survivin shRNA on the Survivin protein expression in SW480 cells

**Figure 3:**
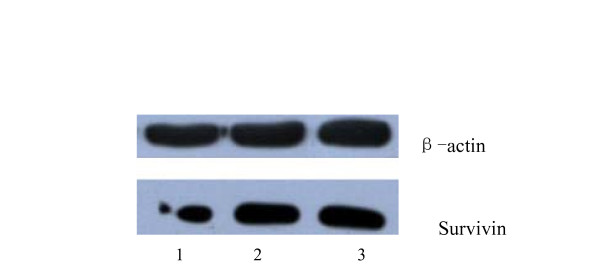
The expression level of Survivin protein of transfected cells.

When analyzed by Western blot, the protein expression levels of the pGCsiRNA-Survivin specific interference group, the negative control group, and the blank control group were 11.32%, 24.08%, and 28.79%, respectively. When the specific interference group was compared to the blank control group, the expression level of Survivin protein was significantly reduced (p < 0.05), with an inhibitory rate of 60.68%. In the comparison between the negative plasmid group and the blank control group, the expression of Survivin had no obvious change (p > 0.05) (Figure 3).

### The influence of pGCH1/Survivin shRNA on the cell cycle and the apoptosis of SW480 cells

**Figure 4:**
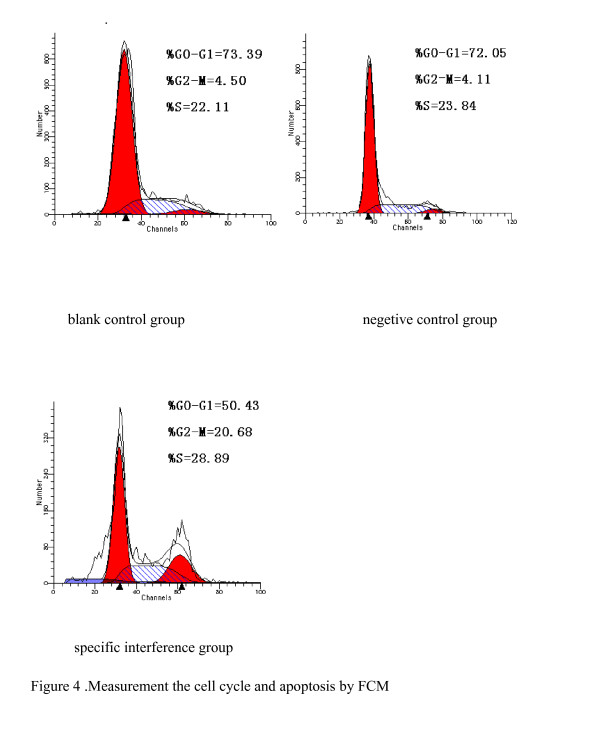
Measurement the cell cycle and apoptosis by FCM.

In the comparison of the specific interference group transfected with pGCsiRNA-Survivin and the blank control group, the percentage of cells in G2/M phase was increased in the specific interference group (P < 0.05). In the comparison of the negative control group and the blank control group, there was no difference seen in the cell cycle. There were no apoptotic peaks in all three groups (Figure 4).

### The invasion ability of SW480 cells tested by Transwell's chamber

**Figure 5:**
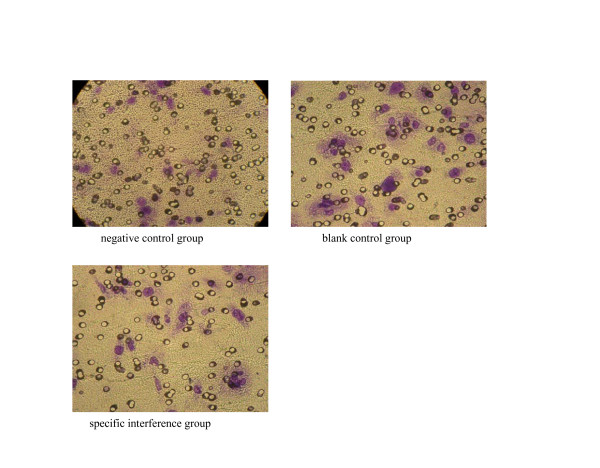
The invasion test of SW480 cells.

The ability of tumor cells to permeate the chambers was significantly reduced in the specific interference group transfected with pGCsiRNA-Survivin. It could be speculated that pGCsi-Survivin could reduce the invasion ability of SW480 cells of colorectal cancer. The inhibitory rate was 44.08% (Figure 5) (See table 1 in additional file 1).

## 4. Discussion

Colorectal cancer has the characteristics of powerful invasion ability and early metastatic property, which are the primary reasons for failure in therapy. To research the molecular mechanisms for invasion and metastasis of colorectal tumor cells, as well as finding treatment target site, has significant meaning for improvement the prognostic outcome. Currently, researches that involved the gene and cytokines such as CD44 and E-cadherin, which were related to tumor metastasis, revealed that the expression level was closely related to the metastatic ability. In the metastatic process, tumor cells would have to fall off from primary lesion and penetrate the basal membrane. Tumor cells had characteristics of being infiltrative, in which it could penetrate the basal membrane to achieve infiltrative metastasis. In this study, the ability of cells to penetrate Matrigel was used as an indicator to reflect the invasion ability.

Survivin gene is generally highly expressed in human colorectal tumor cells, and relates to the decrease in the apoptotic index, shortened the overall survival rate, poor prognosis and the increase in recurrence rate [4-6]. Therefore, Survivin had become an important target for tumor therapy, in which most methods involved the reduction of Survivin expression as a way to treat tumor. The mechanism mostly was inhibiting the expression of Survivin to promote apoptosis of tumor cells, and thus, to inhibit the proliferation of tumor cells [7,8]. In this study, pGCH1/Survivin shRNA was used to transfect colorectal tumor cell SW480, to reduce the expression of Survivin protein. Analysis by flow cytometry revealed that cells were delayed at G2/M phase, but there was no increase in apoptosis, which were similar to other reports [10]. It suggested that Survivin shRNA could, through other mechanisms other than the apoptotic pathway, inhibit the biological activities of tumor. After the expression of Survivin was down-regulated, the invasion ability of SW480 cells was greatly reduced, suggesting that the expression of Survivin was related to the infiltrative property of colorectal tumor cells. The involving mechanism was probably that changes in microtubule structures occurred when cells were delayed at G2/M phase, which further reduced the cellular elasticity coefficient and thus the reduce in invasion ability [11]. In addition, there were reports that the inhibition of Survivin expression could decrease the expression of tumor angiogenesis factor, and further prevented the invasion of SW480 cells [9,12,13].

In this research, it was discovered that the inhibition of Survivin expression could reduce the invasion ability of colorectal tumor cells, suggesting that Survivin gene was related to the tumor metastasis. By RNA interference method to reduce the expression of Survivin, the metastasis of tumor could be inhibited as well. This would provide evidences for further research into the characteristics of Survivin and RNA interference in the aspect of tumor genetic therapy. The interaction between the metastasis of tumor and metastasis-related factors was extremely complicated, and there were still many unknown involving mechanisms, which required further investigation.

## Note

Table 1. The number of invasive cells of each group.

## Supplementary Material

Additional file 1 Click here for file
