# Supplementary material for: The influence of survivin shRNA on the cell cycle and the invasion of SW480 cells of colorectal carcinoma
Source: J Exp Clin Cancer Res. 2008 Jul 18;27(1):20. doi: 10.1186/1756-9966-27-20 (PMC2515282; doi:10.1186/1756-9966-27-20)
Supplement: Additional file 1 [file 1756-9966-27-20-S1.doc]

Table 1. The number of invasive cells of each group

| **Group** | **Penetration numbers** | **Inhibition ratio（%）** |
| --- | --- | --- |
| Blank group | 25.86±7.45 | 0 |
| Negetive control group | 25.12±8.37 | 2.86 |
| *Specific interference group* | 14.46±2.11 | 44.08* |
